# Association between neutrophil-to-albumin ratio and long-term mortality of aneurysmal subarachnoid hemorrhage

**DOI:** 10.1186/s12883-023-03433-x

**Published:** 2023-10-19

**Authors:** Renjie Zhang, Yu Zhang, Zheran Liu, Yiyan Pei, Yan He, Jiayi Yu, Chao You, Lu Ma, Fang Fang

**Affiliations:** 1 Department of Neurosurgery, West China Hospital of Sichuan University, Chengdu, Sichuan China; 2 Center for Evidence Based Medical and Clinical Research, Affiliated Hospital of Chengdu University, Chengdu, Sichuan China; 3 Department of Biotherapy, Cancer Center, West China Hospital of Sichuan University, Chengdu, Sichuan China; 4 School of Medical and Life Sciences, Chengdu University of Traditional Chinese Medicine, Chengdu, Sichuan China

**Keywords:** Intracranial Aneurysm, Subarachnoid Hemorrhage, Prognosis, Neutrophil-to-albumin, Mortality

## Abstract

**Objective:**

The prognosis of aneurysmal subarachnoid hemorrhage (aSAH) survivors is concerning. The goal of this study was to investigate and demonstrate the relationship between the neutrophil-to-albumin ratio (NAR) and long-term mortality of aSAH survivors.

**Methods:**

A retrospective observational cohort study was conducted at Sichuan University West China Hospital between January 2009 and June 2019. The investigation of relationship between NAR and long-term mortality was conducted using univariable and multivariable Cox regression models. To demonstrate the predictive performance of different biomarkers over time, time-dependent receiver operating characteristic curve (ROC) analysis and decision curve analysis (DCA) were created.

**Results:**

In total, 3173 aSAH patients were included in this study. There was a strong and continuous relationship between NAR levels and long-term mortality (HR 3.23 95% CI 2.75–3.79, p < 0.001). After adjustment, the result was still significant (adjusted HR 1.78 95% CI 1.49–2.12). Compared with patients with the lowest quartile (< 0.15) of NAR levels, the risk of long-term mortality in the other groups was higher (0.15–0.20: adjusted HR 1.30 95% CI 0.97–1.73; 0.20–0.28: adjusted HR 1.37 95% CI 1.03–1.82; >0.28: adjusted HR 1.74 95% CI 1.30–2.32). Results in survivors were found to be still robust. Moreover, out of all the inflammatory markers studied, NAR demonstrated the highest correlation with long-term mortality.

**Conclusions:**

A high level of NAR was associated with increased long-term mortality among patients with aSAH. NAR was a promising inflammatory marker for long-term mortality of aSAH.

**Supplementary Information:**

The online version contains supplementary material available at 10.1186/s12883-023-03433-x.

## Introduction

Aneurysmal subarachnoid hemorrhage (aSAH) is a condition characterized by a high mortality rate and substantial disability. The current short-term mortality rate for aSAH has decreased to approximately 25–30% due to advances in medical decision-making [1, 2]. Long-term mortality risk remains 1.5 times that of the general population for individuals who have survived the initial aSAH and attained a favorable recovery at the 12-month mark [3]. The elevated mortality rate persisted for a duration of up to two decades following the occurrence of aSAH [4]. Therefore, the long-term survival of aSAH patients merits more consideration.

In the current studies, many predictive markers were used to determine the short-term risk of mortality in patients with aSAH, but few were used to determine the long-term risk of death [5–7]. Previous research has demonstrated a correlation between systemic inflammation and long-term survival [8, 9]. To demonstrate the systemic inflammatory status, numerous inflammatory markers were calculated, including the neutrophil-to-albumin ratio (NAR), platelet-to-albumin ratio (PAR), neutrophil-to-lymphocyte ratio (NLR), platelet-to-lymphocyte ratio (PLR), monocyte-to-lymphocyte ratio (MLR), and systemic immune inflammation index (SII). It has been reported that these biomarkers are optimistic predictors of long-term survival in other diseases, such as cardiovascular diseases [10–12] and cancers [13–17]. NAR, NLR, PLR, and SII index were discovered to be associated with complications after aSAH, including postoperative pneumonia [18], rebleeding [19], and delayed cerebral ischemia [20–24]. Additionally, NAR [25], NLR [19, 26], PLR [22], MLR [27], and SII index [28] were highly predictive of unfavorable short-term outcomes of aSAH. However, the predictive value of these inflammatory factors for long-term survival in patients with aSAH has not been evaluated. Consequently, it is crucial to identify a biomarker that differentiates the long-term prognosis of aSAH survivors.

Our study aimed to determine whether NAR is associated with aSAH survivors’ long-term prognosis. In addition, we would investigate the NAR’s prognostic ability and compare it to other inflammatory markers.

## Methods

### Study design and data source

Between January 2009 and June 2019, a retrospective observational cohort study was conducted at Sichuan University West China Hospital. The department of information assisted in retrieving hospitalization data via the electronic health record system. The West China Hospital Institutional Review Board approved the trial (No. 20,211,701) in which informed consent is not required due to its observational design. Patients were treated in accordance with established guidelines [29, 30].

### Patient selection

In this investigation, patients with SAH were included and SAH must be caused by a clearly responsible aneurysm. SAH should be confirmed by a CT scan or CT angiography (CTA) or digital subtraction angiography (DSA). To detect aneurysms, DSA, CTA, or MR angiography (MRA) were utilized.

Exclusion criteria: (1) aSAH patients due to non-aneurysmal causes such as trauma, arteriovenous malformations, and arteriovenous fistula; (2) aneurysms treated in other hospitals; (3) patients with necessary biological parameters missing, or (4) patients with incorrect ID numbers or from other provinces.

### Inflammatory markers

The exposure of this study was NAR, one of the inflammatory markers, which was calculated as $NAR=\frac{{N{\text{neutrophil}}}}{{N{\text{albumin}}}}$. The West China Hospital Stroke Unit took peripheral blood samples and evaluated serum neutrophil and albumin levels at the time of admission. Additionally, we collected all platelet, lymphocyte, and monocyte records at admission. In addition to PAR, NLR, PLR, MLR, and SII index, five other combinations of inflammatory markers were examined: PAR, NLR, PLR, and MLR. Figure S1 presents calculation methodologies for the various combinations of inflammatory factors.

### Survival status

In this study, the survival status of patients was retrieved from the household registration system of Sichuan province through ID number. According to state regulations, all residents must register the date of death at the local police station within one month of the time of death. This investigation excluded patients whose home address was not in Sichuan province or whose medical record ID number was incorrect. There were a total of 1,797 patients excluded from the study (28.9%). The median survival of all enrolled patients was 45.6 months and the longest follow-up time was 122.4 months.

### Primary and secondary outcomes

The primary outcome was all-cause mortality within the observation period. Secondary outcomes were all-cause mortality within the first hospitalization or after one year. Long-term and short-term were defined through time. These outcomes were also evaluated in post-discharge survivors.

### Statistical analysis

Analysis of variance (ANOVA) was used to analyze continuous variables with a normal distribution, whereas Kruskal-Wallis or Mann-Whitney U tests were used to compare continuous variables with non-normal distributions. The chi-square test or Fisher’s exact test was used to analyze all categorical variables. A 2-sided p value < 0.05 was considered statistically significant. Missing data from continuous variables were imputed using mean values while missing data from categorical variables were labeled as dummy variables. Size of aneurysm and Fisher grade were the only missing variables, accounting for 23.2% and 27.1%, respectively.

Using a restricted cubic spline (RCS), the relationship between NAR levels and all-cause mortality was visualized. Using Kaplan-Meier curves, the difference between NAR categories was compared. The Cox regression model was used to estimate the adjusted hazard ratio (HR) and 95% confidence interval (95% CI). Before conducting a multivariable Cox analysis, univariable Cox regression was performed. Characteristics that influenced the results (p < 0.10) were then incorporated into a multivariable Cox regression model. When factors remained statistically significant, they were deemed independently associated.

As suggested by the TRIPOD statement, Harrell C-statistics (C-index) and integrated discrimination improvement (IDI) were used to compare the ability of NAR and other inflammatory biomarkers to predict long-term mortality [31, 32]. The IDI is computed by adding the proportion of patients whose probability is forecasted to increase to the proportion of non-patients whose probability is predicted to decrease. If IDI is greater than zero, the new model’s predictive ability is superior to the previous model’s. The clinical usefulness was evaluated using the categorical net reclassification improvement (cNRI) and the decision curve analysis (DCA). cNRI is an index for comparing the classification ability of old and novel models using the gold standard diagnosis. Similar to the IDI, the larger the cNRI, the more apparent the model enhancement. According to the previous study [1], we defined risk ratio < 0.1 as low, 0.1–0.6 as moderate, and > 0.6 as high risk of long-term mortality. To evaluate the change in discriminatory ability of these biomarkers over time, we constructed receiver operating characteristic (ROC) curves at various time points and the time-dependent ROC curve. Higher area under the curve (AUC) indicated a greater capacity for prediction.

Subgroup analysis was employed to determine whether the association between NAR and long-term mortality varied across subgroups, and the p value for interaction was calculated. All analyses were performed using R software (version 4.1.0, Vienna, Austria).

## Results

### Baseline characteristics of the cohort

**Fig. 1:**
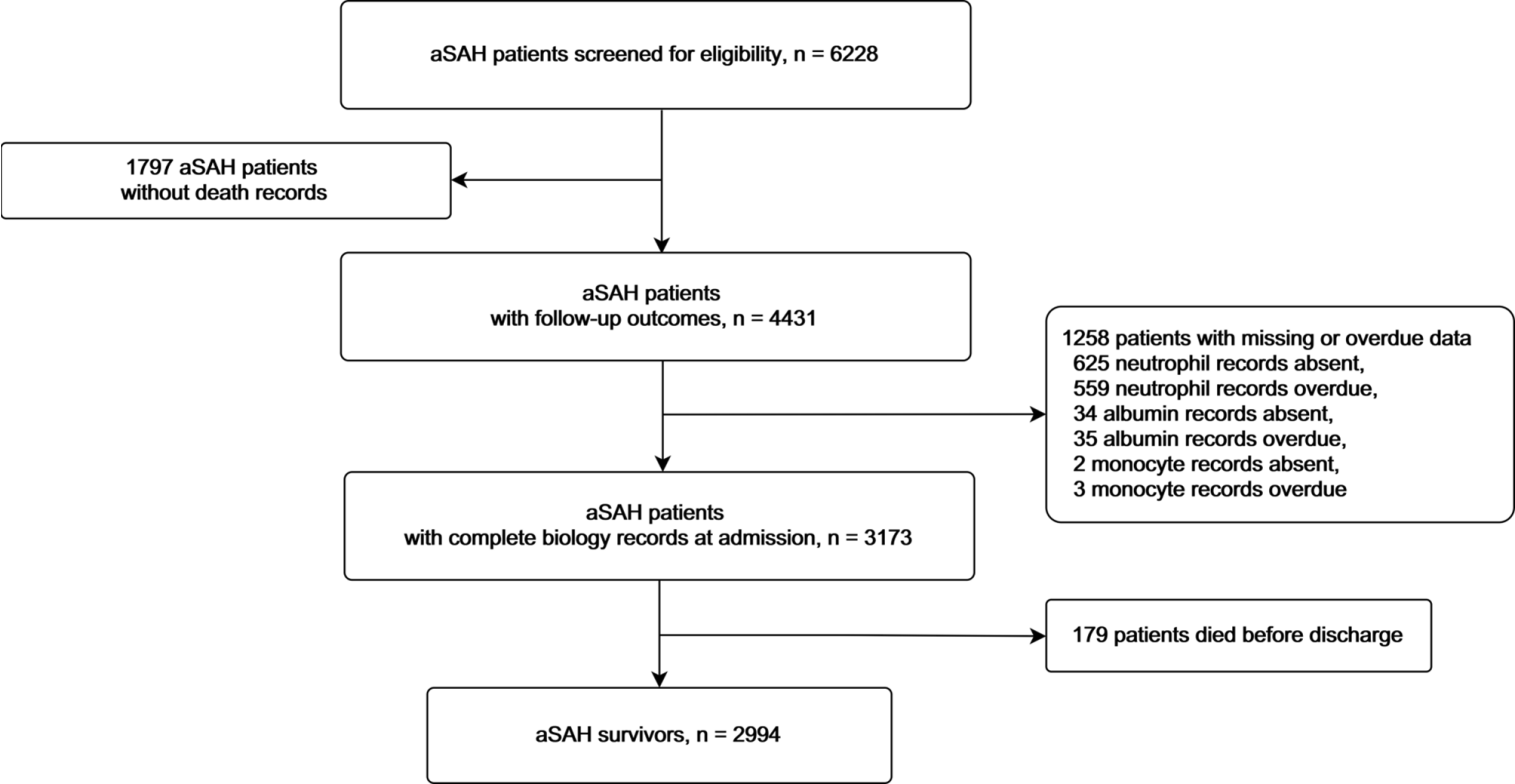
Flow diagram for the selection of participants included in the present analysis

3173 aSAH patients were enrolled in this study (Fig. 1). Table 1 displays the baseline characteristics of the included patients. The mean age of our cohort was 55.14 ± 11.99 years, and 1113 (35.1%) were female. Aneurysms of the posterior circulation were found in 18.9% of patients, with a mean size of 0.77 ± 0.71 cm. Patients with a high NAR (> 0.28) were more likely to have a higher SBP at admittance and a history of hypertension (p < 0.001 and p = 0.05, respectively). In addition, patients with a high NAR had a higher Fisher grade and Hunt-Hess grade (p < 0.001, both).

**Table 1:** Baseline characteristics stratified by NAR levels at admission.

Characteristics	All	NAR quartile				*P* value
	n = 3173	< 0.15 (n = 794)	0.15–0.20 (n = 793)	0.20–0.28 (n = 793)	> 0.28 (n = 793)	
Demographics						
Age, y, mean (SD)	55.14 (11.99)	54.49 (12.51)	55.81 (11.44)	55.39 (11.89)	54.85 (12.06)	0.13
Male, n (%)	1113 (35.1)	230 (29.0)	279 (35.2)	295 (37.2)	309 (39.0)	< 0.001
Smoking, n (%)						
Current	142 (4.5)	44 (5.5)	39 (4.9)	31 (3.9)	28 (3.5)	0.03
Ever	642 (20.2)	131 (16.5)	161 (20.3)	168 (21.2)	182 (23.0)	
Never	2389 (75.3)	619 (78.0)	593 (74.8)	594 (74.9)	583 (73.5)	
Alcohol abuse, n (%)	630 (19.9)	125 (15.7)	164 (20.7)	173 (21.8)	168 (21.2)	0.009
SBP, mmHg, mean (SD)	144.38 (25.12)	137.07 (21.24)	144.86 (23.15)	147.32 (25.35)	148.29 (28.64)	< 0.001
Medical history, n (%)						
Hypertension	773 (24.4)	175 (22.0)	189 (23.8)	188 (23.7)	221 (27.9)	0.05
Diabetes	188 (5.9)	50 (6.3)	39 (4.9)	46 (5.8)	53 (6.7)	0.48
CHD	79 (2.5)	19 (2.4)	16 (2.0)	21 (2.6)	23 (2.9)	0.71
CRF	20 (0.6)	4 (0.5)	5 (0.6)	4 (0.5)	7 (0.9)	0.80
COPD	229 (7.2)	50 (6.3)	70 (8.8)	54 (6.8)	55 (6.9)	0.23
Aneurysm characteristics						
Posterior location, n (%)	601 (18.9)	149 (18.8)	153 (19.3)	155 (19.5)	144 (18.2)	0.90
Size, cm, median (IQR)	0.60 (0.40, 0.80)	0.60 (0.40, 0.90)	0.60 (0.40, 0.80)	0.60 (0.40, 0.80)	0.60 (0.40, 0.80)	0.30
Hemorrhagic characteristics, n (%)						
Fisher grade III-IV	1689 (53.2)	15 (1.9)	44 (5.5)	85 (10.7)	265 (33.4)	< 0.001
Hunt & Hess grade IV-V	409 (12.9)	197 (24.8)	417 (52.6)	478 (60.3)	597 (75.3)	< 0.001
EVD	71 (2.2)	1 (0.1)	12 (1.5)	15 (1.9)	43 (5.4)	< 0.001
Treatment of aneurysms, n (%)						
Clip	2123 (66.9)	505 (63.6)	540 (68.1)	573 (72.3)	505 (63.7)	< 0.001
Coil	395 (12.4)	131 (16.5)	106 (13.4)	77 (9.7)	81 (10.2)	
No treatment	655 (20.6)	158 (19.9)	147 (18.5)	143 (18.0)	207 (26.1)	
Biology, median (IQR)						
Glucose, mmol/L	6.41 (5.46, 7.77)	5.43 (4.86, 6.41)	6.25 (5.50, 7.19)	6.69 (5.81, 7.95)	7.48 (6.29, 9.33)	< 0.001
Neutrophil, 10^9^/L	8.07 (5.68, 11.15)	4.24 (3.16, 5.07)	7.00 (6.34, 7.78)	9.52 (8.58, 10.62)	13.99 (12.16, 16.42)	< 0.001
Platelet, 10^9^/L	165.00 (126.00, 211.00)	163.00 (123.00, 212.00)	157.00 (124.00, 204.00)	169.00 (127.00, 208.00)	169.00 (129.00, 221.00)	0.01
Lymphocyte, 10^9^/L	1.10 (0.78, 1.49)	1.38 (1.02, 1.75)	1.10 (0.82, 1.46)	1.02 (0.68, 1.38)	0.92 (0.67, 1.28)	< 0.001
Albumin, g/L	40.50 (37.10, 43.40)	40.60 (38.10, 43.10)	40.70 (37.50, 43.40)	40.80 (37.30, 43.60)	39.70 (34.50, 43.80)	< 0.001
Monocyte, 10^9^/L	0.48 (0.34, 0.67)	0.37 (0.28, 0.47)	0.46 (0.35, 0.60)	0.54 (0.38, 0.70)	0.66 (0.48, 0.91)	< 0.001
NAR	0.20 (0.15, 0.28)	0.11 (0.08, 0.13)	0.17 (0.16, 0.19)	0.24 (0.22, 0.26)	0.35 (0.31, 0.41)	< 0.001
PAR	4.11 (3.14, 5.32)	4.06 (3.03, 5.31)	3.96 (3.07, 5.02)	4.12 (3.22, 5.29)	4.40 (3.30, 5.70)	< 0.001
NLR	7.54 (4.24, 12.87)	2.85 (1.98, 4.32)	6.29 (4.64, 8.93)	9.41 (6.62, 13.89)	15.17 (10.52, 22.44)	< 0.001
PLR	148.26 (105.83, 215.48)	118.05 (90.72, 167.31)	141.49 (104.52, 203.57)	164.75 (118.10, 231.43)	178.69 (121.54, 263.54)	< 0.001
MLR	0.44 (0.29, 0.66)	0.26 (0.20, 0.37)	0.41 (0.30, 0.54)	0.50 (0.35, 0.71)	0.69 (0.47, 1.00)	< 0.001
SII	1.19 (0.67, 2.10)	0.48 (0.33, 0.73)	0.99 (0.71, 1.44)	1.57 (1.09, 2.24)	2.52 (1.62, 4.03)	< 0.001

SBP, systolic blood pressure; CHD, coronary heart disease; COPD, chronic obstructive pulmonary disease; CRF, chronic renal failure; EVD, external ventricular drain; SD, standard deviation; NAR, neutrophil-to-albumin ratio; PAR, platelet-to-albumin ratio; NLR, neutrophil-to-lymphocyte ratio; PLR, platelet-to-lymphocyte ratio; MLR, monocyte-to-lymphocyte ratio; SII, systemic immune inflammation index

### Association between NAR and survival

**Fig. 2:**
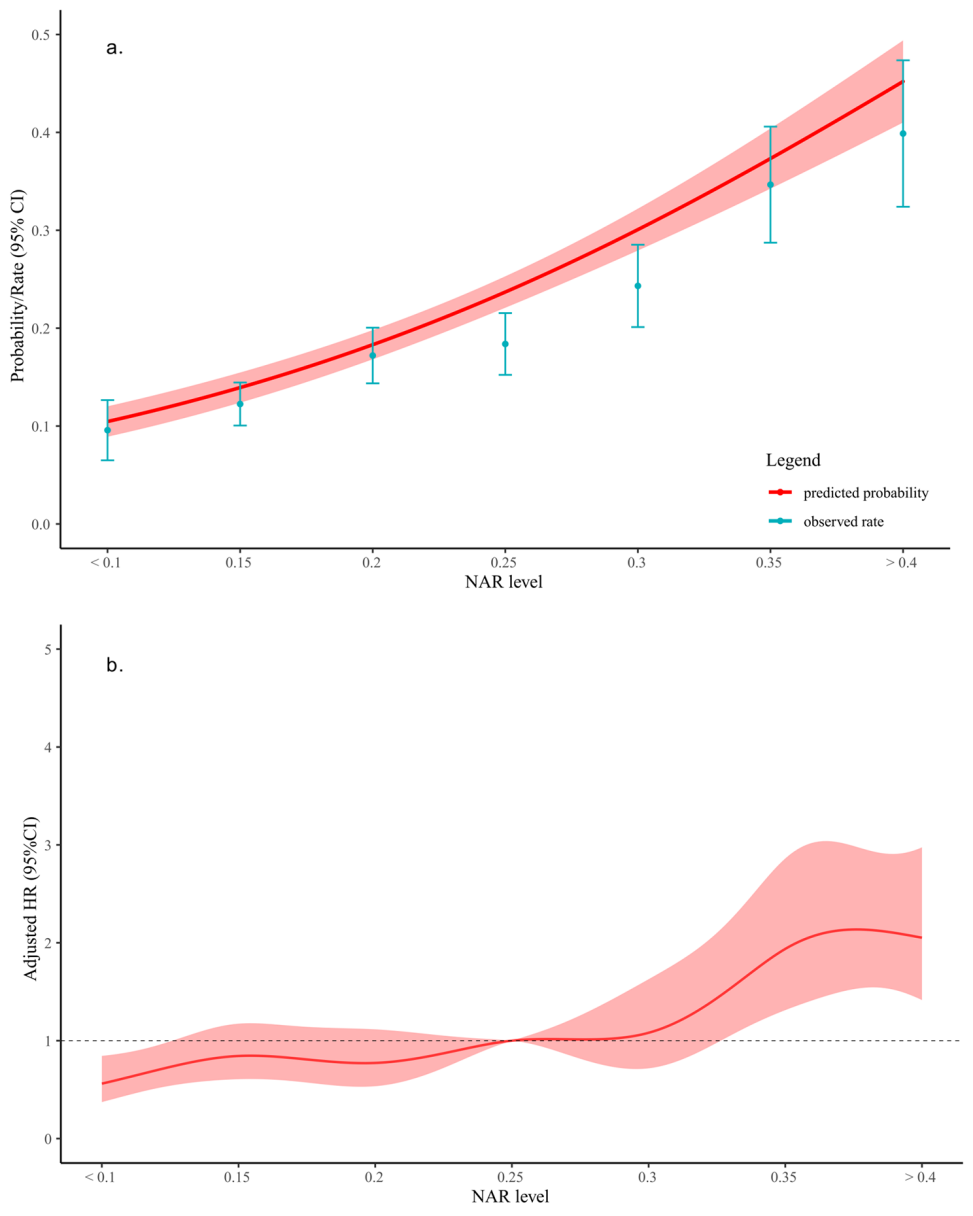
Relationship between NAR and long-term mortality in all patients with aSAH. Predicted probabilities and observed rate of long-term mortality (**a**). Adjusted HR and 95% CI are shown for each 0.05 change (**b**). NAR, neutrophil-to-albumin ratio; aSAH, aneurysmal subarachnoid hemorrhage; HR, hazard ratio; CI, confidence interval

As demonstrated in Fig. 2 and Figure S2, there was a robust and continuous association between NAR levels and long-term mortality (HR 3.23 95% CI 2.75–3.79, p < 0.001) and long-term mortality in discharged survivors (HR 2.52 95% CI 2.10–3.03, p < 0.001). After accounting for all potential confounders, the result remained statistically significant (adjusted HR 1.78 95% CI 1.49–2.12; adjusted HR 1.55 95% CI 1.26–1.90, respectively).

**Fig. 3:**
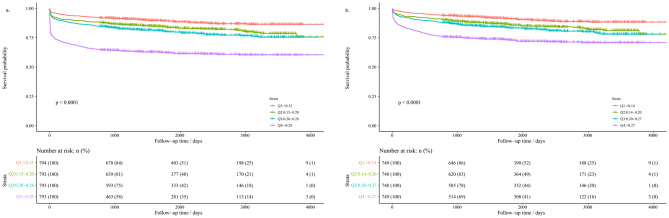
Kaplan-Meier curve for overall survival of all patients (n = 3173) by quartiles of NAR levels (**a**). Kaplan-Meier curve for overall survival of discharged survivors (n = 2994) by quartiles of NAR levels (**b**). NAR, neutrophil-to-albumin ratio

Kaplan-Meier curves for all patients (n = 3173) and discharged survivors (n = 2994) were statistically analyzed, as shown in Fig. 3. Among all patients, the median survival time for each NAR group was 68.3 months in Q1 NAR levels (< 0.15), 63.1 months in Q2 NAR levels (0.15–0.20), 57.4 months in Q3 NAR levels (0.20–0.28), and 46.4 months in Q4 NAR levels (> 0.28). There was a significant survival difference between the four groups (p < 0.001). Among the discharged survivors, the median survival time for each NAR group was 70.1 months in Q1 NAR levels (< 0.14), 63.1 months in Q2 NAR levels (0.14–0.20), 64.9 months in Q3 NAR levels (0.20–0.27), and 56.1 months in Q4 NAR levels (> 0.27). There was still a significant survival difference between the four groups (p < 0.001).

The results of the Cox regression model are shown in Table S1. In the univariate Cox regression, factors including age, sex, SBP at admission, history of hypertension, diabetes, CRF, and COPD, location and size of aneurysms, Fisher grade, Hunt & Hess grade, external ventricular drain (EVD), treatment, glucose and NAR levels were implemented into the further analysis. After adjustment, NAR (> 0.28 vs. < 0.15) was an independent prognostic factor (adjusted HR 1.74 95% CI 1.30–2.32, p < 0.001). Besides, age, history of CRF (adjusted HR 2.53 95% CI 1.91–3.15, p = 0.003), size of aneurysms (adjusted HR 1.17 95% CI 1.10–1.25, p < 0.001), Fisher grade (adjusted HR 1.35 95% CI 1.13–1.58, p = 0.008), Hunt & Hess grade (adjusted HR 2.58 95% CI 2.39–2.77, p < 0.001), EVD, treatment and glucose were still statistically significant in the multivariable model.

There were 179 patients (5.6%) who died before discharge. Excluding from the entire cohort, we made a subgroup analysis of the remaining 2994 patients. As shown in Table S2, the same factors as before were employed in the multivariable Cox regression model. After adjustment, NAR (> 0.27 vs. < 0.14) was still significant (adjusted HR 1.55 95% CI 1.11–2.15, p = 0.009). Compared with the results of all patients, a higher Fisher grade (III-IV) was no longer an independent prognostic factor but a higher Hunt & Hess grade (IV-V) was still significant (Fisher grade: adjusted HR 1.19 95% CI 0.95–1.43, p = 0.15; Hunt & Hess grade: adjusted HR 2.30 95% CI 2.07–2.53, p < 0.001). Besides, SBP at admission was still significant in this multivariable model (p = 0.008).

Association between NAR and 1-year mortality was also explored. As shown in Table 2, higher NAR levels (> 0.28 vs. < 0.15) were associated with 1-year mortality (adjusted HR 2.44 95% CI 1.69–3.52, p < 0.001). The result was still robust after excluding patients who died before discharge (NAR > 0.27 vs. < 0.14: adjusted HR 2.37 95% CI 1.50–3.73, p < 0.001). Furthermore, after adjusting for potential confounders, positive dose-response relationships were found between NAR levels and long-term mortality and 1-year mortality (both p for trend < 0.001). Similar dose-response relationships between long-term mortality and NAR were also found in the discharged survivors.

**Table 2:** Associations between NAR levels and mortality in all patients and discharged survivors

Outcomes	NAR levels	Unadjusted			Multivariable Regression Adjustment		
		Unadjusted HR	*P* value	*P* trend	Adjusted HR	*P* value	*P* trend
Mortality before discharge	0.15–0.20	1.24 (0.65–2.37)	0.51	< 0.001	1.11 (0.55–2.25)	0.77	< 0.001
	0.20–0.28	1.55 (0.83–2.88)	0.17		0.86 (0.41–1.80)	0.68	
	> 0.28	7.75 (4.61–13.03)	< 0.001		2.60 (1.36–4.96)	0.004	
1-year mortality	0.15–0.20	1.64 (1.15–2.34)	0.006	< 0.001	1.30 (0.89–1.91)	0.17	< 0.001
	0.20–0.28	2.06 (1.46–2.89)	< 0.001		1.30 (0.89–1.89)	0.17	
	> 0.28	5.79 (4.27–7.84)	< 0.001		2.44 (1.69–3.52)	< 0.001	
Long-term mortality	0.15–0.20	1.55 (1.19–2.02)	0.001	< 0.001	1.30 (0.97–1.73)	0.08	< 0.001
	0.20–0.28	1.92 (1.49–2.48)	< 0.001		1.37 (1.03–1.82)	0.03	
	> 0.28	4.06 (3.22–5.13)	< 0.001		1.74 (1.30–2.32)	< 0.001	
1-year mortality of discharged survivors	0.14–0.20	1.76 (1.14–2.72)	0.011	< 0.001	1.31 (0.81–2.12)	0.26	< 0.001
	0.20–0.27	2.29 (1.51–3.48)	< 0.001		1.55 (0.98–2.46)	0.06	
	> 0.27	4.93 (3.36–7.23)	< 0.001		2.37 (1.50–3.73)	< 0.001	
Long-term mortality of discharged survivors	0.14–0.20	1.63 (1.21–2.19)	0.001	< 0.001	1.34 (0.97–1.85)	0.08	< 0.001
	0.20–0.27	1.96 (1.47–2.62)	< 0.001		1.51 (1.09–2.09)	0.01	
	> 0.27	3.28 (2.50–4.28)	< 0.001		1.55 (1.11–2.15)	0.009	

NAR, neutrophil-to-albumin ratio; HR, hazard rate; CI, confidence interval

### Promising biomarker of aSAH survival

**Fig. 4:**
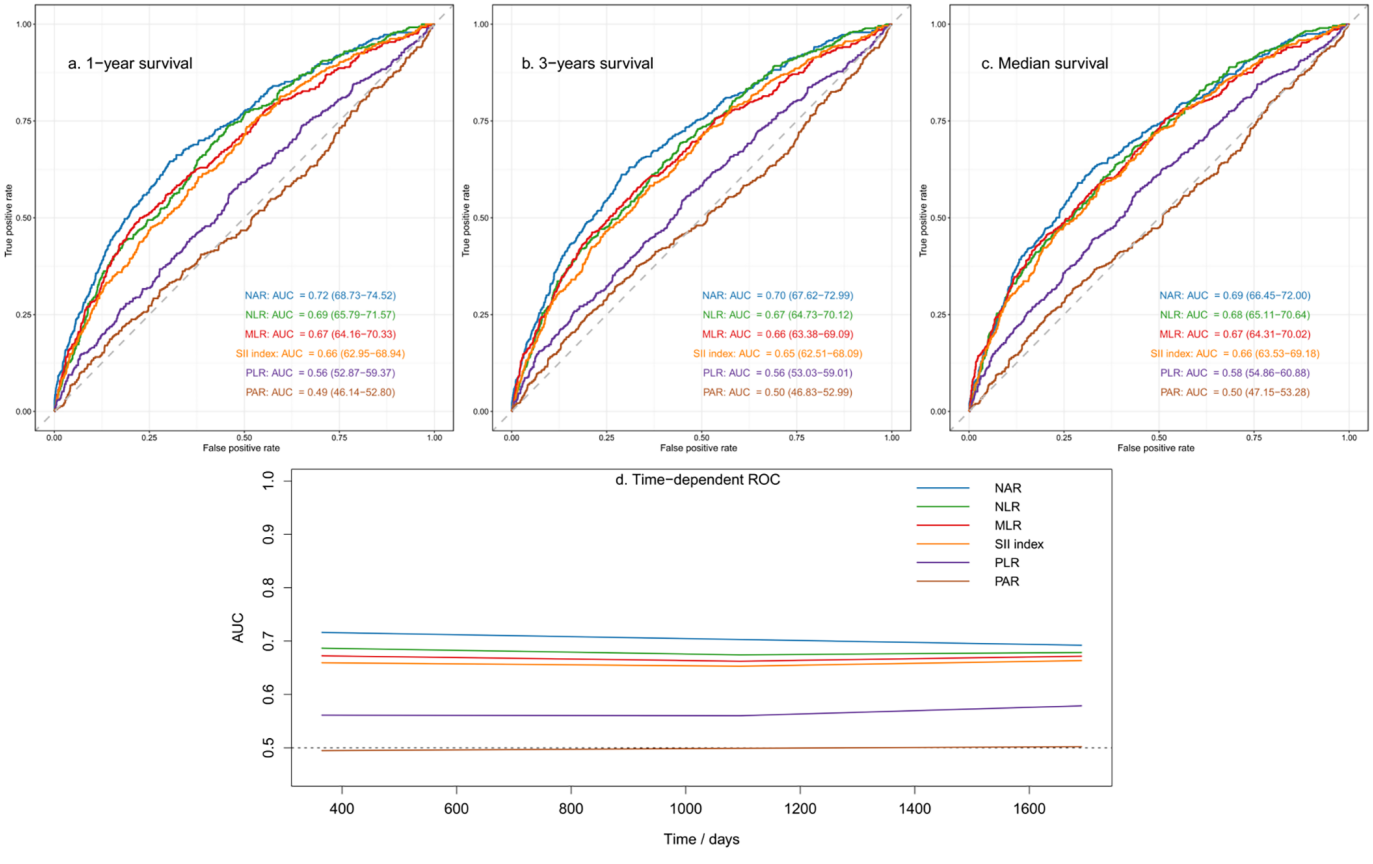
The receiver operating characteristic curves (ROC) of the different inflammatory markers to predict 1-, 3-, and median overall survival (**a-c**). Time-dependent ROC analysis of the predictive accuracy of the different inflammatory markers (**d**). NAR, neutrophil-to-albumin ratio; PAR, platelet-to-albumin ratio; NLR, neutrophil-to-lymphocyte ratio; PLR, platelet-to-lymphocyte ratio; MLR, monocyte-to-lymphocyte ratio; SII index, systemic immune inflammation index; AUC, area under the curve

**Fig. 5:**
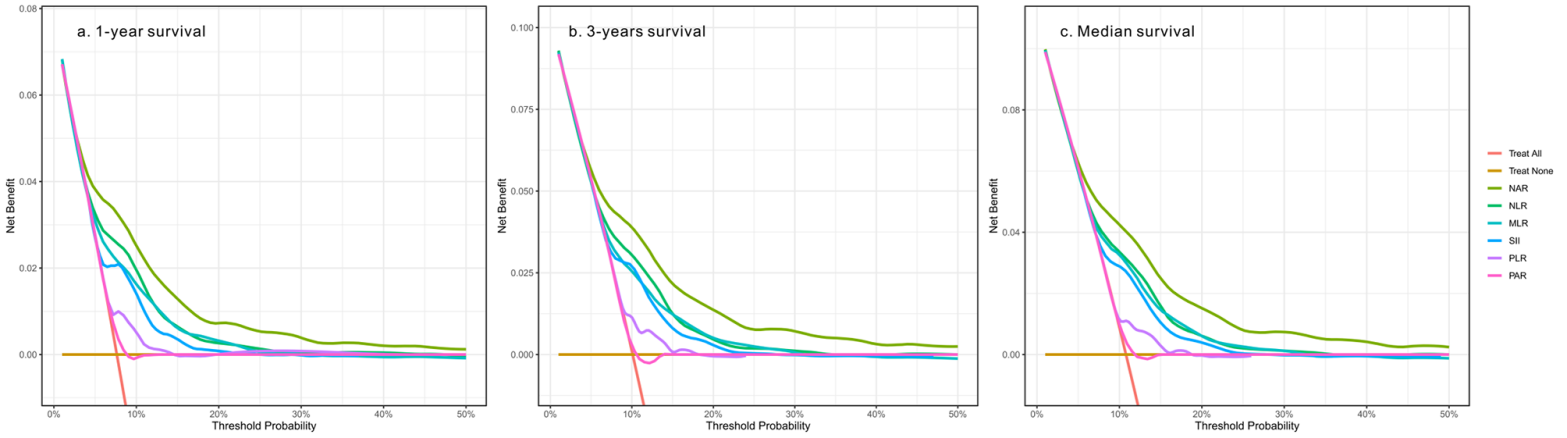
The decision curve analysis (DCA) of the different inflammatory markers to predict 1-, 3-, and median overall survival

C-index, categorical NRI, and IDI were calculated to compare the predictive efficacy of NAR to that of other inflammatory biomarkers (Table 3). The best cut-off value of NAR for predicting long-term mortality was 0.25 with 53.9% sensitivity and 71.3% specificity (AUC = 0.68 95% CI: 0.65–0.70). Compared with the five other inflammatory biomarkers, NAR achieved the highest C-index (C-index = 0.68 95% CI: 0.66–0.70). In addition, as shown in Table S3, NAR demonstrated a superior capacity for reclassification (cNRI: p < 0.001, all). Furthermore, time-dependent ROC curves were generated for each biomarker, and the estimated AUCs were calculated at different time points (Fig. 4). The prognostic performance of NAR was continuously superior to other inflammatory biomarkers. As shown in Fig. 5, NAR showed a higher net benefit consistently in the DCA of 3 time points, which means more promising clinical application prospects.

**Table 3:** Discriminative capacity of NAR and other inflammatory biomarkers to predict long-term mortality

Biomarkers	C-index (95% CI)	*P* value	cNRI (95% CI)	*P* value	IDI (95% CI)	*P* value
NAR vs. PAR	0.68 (0.66–0.70) vs. 0.50 (0.47–0.53)	< 0.001	0.06 (0.04–0.09)	< 0.001	0.04 (0.03–0.05)	< 0.001
NAR vs. NLR	0.68 (0.66–0.70) vs. 0.65 (0.63–0.68)	0.004	0.07 (0.04–0.09)	< 0.001	0.04 (0.02–0.05)	< 0.001
NAR vs. PLR	0.68 (0.66–0.70) vs. 0.55 (0.53–0.58)	< 0.001	0.07 (0.05–0.09)	< 0.001	0.05 (0.04–0.06)	< 0.001
NAR vs. MLR	0.68 (0.66–0.70) vs. 0.64 (0.62–0.67)	0.014	0.07 (0.05–0.09)	< 0.001	0.07 (0.05–0.08)	< 0.001
NAR vs. SII	0.68 (0.66–0.70) vs. 0.64 (0.61–0.66)	< 0.001	0.07 (0.05–0.09)	< 0.001	0.08 (0.06–0.09)	< 0.001

NAR, neutrophil-to-albumin ratio; PAR, platelet-to-albumin ratio; NLR, neutrophil-to-lymphocyte ratio; PLR, platelet-to-lymphocyte ratio; MLR, monocyte-to-lymphocyte ratio; SII, systemic immune inflammation index; cNRI, categorical net reclassification improvement; IDI, integrated discrimination improvement; CI, confidence interval

We also evaluated the interactions between various factors and NAR (Figure S3). There was an interaction between age and COPD history (p = 0.01, p = 0.02, respectively). Fisher grade III-IV, Hunt & Hess grade IV-V, aneurysm therapy, or aneurysm site had no discernible impact on the link between NAR and death.

## Discussion

In this extensive retrospective observational study, we demonstrated that a high level of NAR is associated with an increased risk of long-term mortality among aSAH survivors. In addition, we discovered a dose-response relationship between elevated NAR levels and long-term mortality. In addition, we demonstrated that NAR was a promising inflammatory biomarker for the prediction of long-term aSAH mortality using a multidimensional approach.

Although there have been numerous studies on the long-term prognosis of aSAH, their definition of long-term prognosis was typically one year after aSAH [33–37]. However, the overburdened risk of mortality in patients with aSAH persists for much longer than one year, necessitating urgent studies on the prognosis of aSAH. Several studies have revealed the association between NAR and complications and 3-months poor outcomes [18, 20, 25, 38]. In the absence of long-term follow-up, however, the predictive value of NAR for the long-term prognosis of aSAH was null. The association of NAR with the early prognosis of aSAH was primarily attributable to the inflammatory response induced by early brain injury (EBI), although it was unknown whether this effect would persist over time. We conducted a comprehensive investigation of the association between NAR and aSAH long-term mortality using data from 3,173 aSAH patients with a median follow-up period of 3.8 years.

In this study, we compared NAR to other inflammatory markers and found that NAR was a more accurate predictor of long-term prognosis. First, the NAR is a simple and inexpensive indicator of inflammation that can be calculated from the patient’s routine admission measurements without additional financial burden. By analyzing their C-index, we discovered that NAR was more discriminating than other inflammatory markers (p < 0.05, all). In terms of the time-dependent ROC analysis, the NAR prediction was more stable than those of the other markers, which reflected the stability of its long-term predictive ability. Thirdly, we found that NAR was superior to DCA and categorical NRI in identifying high-risk (60%) aSAH patients. This means that we could identify aSAH survivors with a high long-term risk earlier and focus more on follow-up and early intervention. More importantly, NAR suggested therapeutic directions that were more likely to be pursued in clinical practice than other inflammatory markers.

Neutrophil depletion after subarachnoid hemorrhage has been reported to improve memory via NMDA receptors [39]. Albumin was not only an important inflammatory marker, but also an important nutritional marker. Albumin supplementation in perioperative patients has long been a cause for concern. A multicenter pilot study also revealed that 1.25 g/kg/day albumin treatment was safe in SAH patients and might be neuroprotective [40].

Complex and diverse pathophysiological mechanisms underlie the association between NAR and mortality. Elevated neutrophil levels were proved to be related to poorer outcomes and in-hospital complications in aSAH [41]. Damage to the brain-blood barrier (BBB) is mediated by neutrophil products, such as free oxygen radicals and proteolytic enzymes [42]. Inflammation is exacerbated by the release of neutrophil extracellular traps, which compromise the blood-brain barrier (BBB) and exacerbate injury to surrounding neurons and other brain cells [43, 44].

In addition, although the precise mechanism is unknown, hypoalbuminemia has been linked to in-hospital complications [45] and outcomes at discharge in aSAH patients [46]. Several experimental studies have reported that albumin exerts a neuroprotective effect by preventing BBB disruption [47], alleviating neural degeneration and apoptosis [48], and assisting neurovascular remodeling [49]. Nevertheless, a previous experiment research indicated that TGF-beta receptor-mediated albumin uptake into astrocytes is involved in neocortical epileptogenesis [50]. To determine the function of albumin in the brain, therefore, larger, more rigorous clinical studies are required.

Intriguingly, in the subgroup analysis, higher NAR levels were associated with long-term mortality in patients ≤ 65 years but not in those > 65 years (p = 0.01). Similar results were found that high-NAR patients without a history of COPD were associated with long-term mortality (p = 0.02). Several studies suggest that neutrophil defensins and serine proteinases cause damage in COPD and stimulate epithelial cells to recruit more neutrophils, thereby exacerbating the inflammatory response [51, 52]. A systematic review found that stable COPD patients have substantially reduced serum albumin concentrations than non-COPD controls [53]. As both neutrophil and albumin levels are positively correlated with COPD severity, NAR may not be an excellent indicator in patients with COPD. Additionally, as previously reported, age-related changes in the local inflamed tissues could cause aberrant neutrophil trafficking and subsequent remote organ damage [54]. Low albumin levels have been linked to older physical endurance levels. It was hypothesized that an increased level of NAR in the elderly could impact the capacity of NAR [55]. While the mechanism is unknown, the subgroup should be read with caution because false-positive findings might occur when many subgroups are examined [56].

Posterior circulation and increased blood volume always portend a poorer prognosis in the short term. We also test these conclusions with our results. The median survival time of patients with posterior circulation aneurysms was significantly shorter than that of patients with anterior circulation aneurysms (1288.13 ± 871.62 vs. 1944.33 ± 1167.88, p < 0.001). Fisher grade was used to measure the blood volume. Patients with higher Fisher grades had shorter survival times than those with lower Fisher grades (1601.52 ± 1129.16 vs. 1963.56 ± 1025.17, p < 0.001). To further examine if they would impact the outcomes, the interaction between these two variables and NAR was calculated. As depicted in Figure S3, there was no interaction between NAR and aneurysm location (p = 0.60) and aSAH patients with higher NAR were at higher risk of long-term death in patients with both anterior circulation (HR = 1.55, 95% CI 1.28–1.88) and posterior location aneurysms(HR = 2.12, 95% CI 1.44–3.12). Similarly, there was no interaction between NAR and Fisher grade (p = 0.91) and aSAH patients with higher NAR were at higher risk of long-term death in patients with both lower Fisher grade (HR = 1.57, 95% CI 1.02–2.40) and higher Fisher grade(HR = 1.66, 95% CI 1.33–2.08). Besides, to avoid the influence of short-term prognosis resulting from the aneurysm location and blood volume, we excluded the patients dead before discharge. As shown in Table S2, the aneurysm location (p = 0.66) and blood volume (p = 0.15) were not associated with long-term mortality after multivariable regression adjustment. In summary, we didn’t consider the location of aneurysm or the blood volume would impact our conclusions.

Our study’s merits are its extensive data and accurate and exhaustive long-term follow-up. In addition, we compared the predictive ability of six inflammatory markers using multiple methodologies.

Nevertheless, our study has several limitations. First, the medication history was absent. Particular medications could affect the neutrophil and albumin levels. Our study was unable to assess this relationship; however, using neutrophil and albumin levels at admission could mitigate this issue. Furthermore, we had no access to the specific causes of death of the survivors. This limits our further exploration of the relationship between inflammation and specific disease risk, such as cardiovascular diseases and cancer. In addition, neither the daily nor long-term NAR levels were measured. Dynamic monitoring of NAR levels would be more beneficial, and long-term NAR levels could indicate the presence of a persistent, long-term, chronic inflammatory condition.

In spite of the aforementioned limitations, our study revealed that NAR may be a novel, cost-effective, and accessible biomarker for predicting the long-term mortality of aSAH patients.

## Conclusion

Our study indicated that a high level of NAR was associated with increased long-term mortality among patients with aSAH and the NAR was a promising inflammatory marker for long-term mortality of aSAH. It may help us better comprehend the connection between inflammatory response and long-term prognosis of aSAH and predict long-term risk of aSAH.

### Electronic supplementary material

Below is the link to the electronic supplementary material.

Supplementary Material 1

## Data Availability

The datasets used and/or analysed during the current study available from the corresponding author on reasonable request.

## References

[1] Macdonald RL, Schweizer TA (2017). Spontaneous Subarachnoid Haemorrhage. The Lancet.

[2] Galea JP, Dulhanty L, Patel HC, Uk, Ireland Subarachnoid Hemorrhage Database C (2017). Predictors of Outcome in Aneurysmal Subarachnoid Hemorrhage patients: observations from a Multicenter Data Set. Stroke.

[3] Huhtakangas J, Lehto H, Seppa K, Kivisaari R, Niemela M, Hernesniemi J, Lehecka M (2015). Long-term excess mortality after Aneurysmal Subarachnoid Hemorrhage: patients with multiple aneurysms at risk. Stroke.

[4] Nieuwkamp DJ, de Wilde A, Wermer MJ, Algra A, Rinkel GJ (2014). Long-term outcome after aneurysmal subarachnoid hemorrhage-risks of vascular events, death from cancer and all-cause death. J Neurol.

[5] Zuo Z, Ji X (2019). Prognostic value of copeptin in patients with aneurysmal subarachnoid Hemorrhage. J Neuroimmunol.

[6] Zheng S, Zhang Y, Wang H, Xie X, Lin Y, Yao P, Lin Z, Kang D. Serum lactate dehydrogenase to phosphate ratio as an Independent predictor for adverse outcome of Microsurgical Clipping for ruptured intracranial Aneurysm: a propensity-score matching analysis. Brain Sci 2022, 12(6). 10.3390/brainsci12060737 PMC9220933 35741622

[7] Cavalli I, Stella C, Stoll T, Mascia L, Salvagno M, Coppalini G, Diosdado A, Menozzi M, Diaferia D, Ndieugnou Djangang N (2023). Serum LDH levels may predict poor neurological outcome after aneurysmal subarachnoid Hemorrhage. BMC Neurol.

[8] Zaccherini G, Tufoni M, Iannone G, Caraceni P. Management of Ascites in patients with Cirrhosis: an update. J Clin Med 2021, 10(22). 10.3390/jcm10225226 PMC8621554 34830508

[9] Sciaccaluga C, Ghionzoli N, Mandoli GE, D’Ascenzi F, Focardi M, Valente S, Cameli M. Biomarkers in patients with left ventricular assist device: an insight on current evidence. Biomolecules 2022, 12(2). 10.3390/biom12020334 PMC8869703 35204834

[10] He HM, Zhang SC, He C, You ZB, Luo MQ, Lin MQ, Lin XQ, Zhang LW, Lin KY, Guo YS (2022). Association between neutrophil percentage-to-albumin ratio and contrast-associated acute kidney injury in patients without chronic Kidney Disease undergoing percutaneous coronary intervention. J Cardiol.

[11] Shen H, Dai Z, Wang M, Gu S, Xu W, Xu G, Liu X (2019). Preprocedural Neutrophil to albumin ratio predicts In-Stent restenosis following carotid angioplasty and stenting. J Stroke Cerebrovasc Dis.

[12] Urbanowicz T, Michalak M, Olasińska-Wiśniewska A, Rodzki M, Witkowska A, Gąsecka A, Buczkowski P, Perek B, Jemielity M. Neutrophil Counts, Neutrophil-to-Lymphocyte Ratio, and Systemic Inflammatory Response Index (SIRI) Predict Mortality after Off-Pump Coronary Artery Bypass Surgery. *Cells* 2022, 11(7). 10.3390/cells11071124 PMC8997598 35406687

[13] Starzer AM, Steindl A, Mair MJ, Deischinger C, Simonovska A, Widhalm G, Gatterbauer B, Dieckmann K, Heller G, Preusser M (2021). Systemic inflammation scores correlate with survival prognosis in patients with newly diagnosed brain metastases. Br J Cancer.

[14] Pikuła A, Skórzewska M, Pelc Z, Mlak R, Gęca K, Sędłak K, Ciseł B, Kwietniewska M, Rawicz-Pruszyński K, Polkowski WP. Prognostic Value of Systemic Inflammatory Response Markers in Patients Undergoing Neoadjuvant Chemotherapy and Gastrectomy for Advanced Gastric Cancer in the Eastern European Population. *Cancers (Basel)* 2022, 14(8). 10.3390/cancers14081997 PMC9029795 35454903

[15] Ma C, Li R, Yu R, Guo J, Xu J, Yuan X, Guo J (2022). Predictive value of preoperative platelet-to-albumin ratio and apolipoprotein B-to-apolipoprotein A1 ratio for osteosarcoma in children and adolescents: a retrospective study of 118 cases. BMC Cancer.

[16] Liu D, Czigany Z, Heij LR, Bouwense SAW, van Dam R, Lang SA, Ulmer TF, Neumann UP, Bednarsch J. The value of platelet-to-lymphocyte ratio as a prognostic marker in Cholangiocarcinoma: a systematic review and Meta-analysis. Cancers (Basel) 2022, 14(2). 10.3390/cancers14020438 PMC8773915 35053599

[17] Ke ZB, Chen H, Chen JY, Cai H, Lin YZ, Sun XL, Huang JB, Zheng QS, Wei Y, Xue XY (2021). Preoperative abdominal fat distribution and systemic immune inflammation were associated with response to intravesical Bacillus Calmette-Guerin immunotherapy in patients with non-muscle invasive Bladder cancer. Clin Nutr.

[18] Zhang X, Zhang S, Wang C, Liu R, Li A (2022). High neutrophil-to-albumin ratio predicts postoperative Pneumonia in Aneurysmal Subarachnoid Hemorrhage. Front Neurol.

[19] de Morais A, Ribeiro Baylão VM, Martins Silva T, Gomes Dos Santos A, Azevedo M, Nóbrega Lima Rodrigues (2021). A JMdO: is neutrophil-lymphocyte ratio a useful tool for predicting outcome in subarachnoid Hemorrhage? A systematic review. Neurosurg Rev.

[20] Zhang X, Liu Y, Zhang S, Wang C, Zou C, Li A (2021). Neutrophil-to-albumin ratio as a biomarker of delayed cerebral ischemia after Aneurysmal Subarachnoid Hemorrhage. World Neurosurg.

[21] Bolton WS, Gharial PK, Akhunbay-Fudge C, Chumas P, Mathew RK, Anderson IA (2022). Day 2 neutrophil-to-lymphocyte and platelet-to-lymphocyte ratios for prediction of delayed cerebral ischemia in subarachnoid Hemorrhage. Neurosurg Focus.

[22] Tao C, Wang J, Hu X, Ma J, Li H, You C (2017). Clinical value of neutrophil to lymphocyte and platelet to lymphocyte ratio after Aneurysmal Subarachnoid Hemorrhage. Neurocrit Care.

[23] Geraghty JR, Lung TJ, Hirsch Y, Katz EA, Cheng T, Saini NS, Pandey DK, Testai FD (2021). Systemic Immune-inflammation index predicts delayed cerebral vasospasm after Aneurysmal Subarachnoid Hemorrhage. Neurosurgery.

[24] Chen L, Pandey S, Shen R, Xu Y, Zhang Q (2021). Increased systemic Immune-inflammation index is Associated with delayed cerebral ischemia in Aneurysmal Subarachnoid Hemorrhage patients. Front Neurol.

[25] Zhang X, Zhang S, Wang C, Li A (2022). Neutrophil-to-albumin ratio as a novel marker predicting unfavorable outcome in aneurysmal subarachnoid Hemorrhage. J Clin Neurosci.

[26] Shi M, Yang C, Tang QW, Xiao LF, Chen ZH, Zhao WY (2021). The Prognostic Value of Neutrophil-to-lymphocyte ratio in patients with Aneurysmal Subarachnoid Hemorrhage: a systematic review and Meta-analysis of Observational studies. Front Neurol.

[27] Feghali J, Kim J, Gami A, Rapaport S, Caplan JM, McDougall CG, Huang J, Tamargo RJ, Jackson CM (2021). Monocyte-based inflammatory indices predict outcomes following aneurysmal subarachnoid Hemorrhage. Neurosurg Rev.

[28] Yun S, Yi HJ, Lee DH, Sung JH (2021). Systemic inflammation response index and systemic Immune-inflammation index for Predicting the prognosis of patients with Aneurysmal Subarachnoid Hemorrhage. J Stroke Cerebrovasc Dis.

[29] Connolly ES, Rabinstein AA, Carhuapoma JR, Derdeyn CP, Dion J, Higashida RT, Hoh BL, Kirkness CJ, Naidech AM, Ogilvy CS (2012). Guidelines for the management of aneurysmal subarachnoid Hemorrhage: a guideline for healthcare professionals from the American Heart Association/american Stroke Association. Stroke.

[30] Dong Y, Guo ZN, Li Q, Ni W, Gu H, Gu YX, Dong Q (2019). Chinese Stroke Association guidelines for clinical management of cerebrovascular disorders: executive summary and 2019 update of clinical management of spontaneous Subarachnoid Haemorrhage. Stroke Vasc Neurol.

[31] Patzer RE, Kaji AH, Fong Y (2021). TRIPOD reporting guidelines for Diagnostic and Prognostic studies. JAMA Surg.

[32] Heus P, Reitsma JB, Collins GS, Damen J, Scholten R, Altman DG, Moons KGM, Hooft L. Transparent Reporting of Multivariable Prediction Models in Journal and Conference Abstracts: TRIPOD for Abstracts. *Ann Intern Med* 2020. 10.7326/M20-0193 32479165

[33] Wong GK, Lam SW, Wong A, Mok V, Siu D, Ngai K, Poon WS (2014). Early MoCA-assessed cognitive impairment after aneurysmal subarachnoid Hemorrhage and relationship to 1-year functional outcome. Transl Stroke Res.

[34] Nylén K, Csajbok LZ, Ost M, Rashid A, Karlsson JE, Blennow K, Nellgård B, Rosengren L (2006). CSF -neurofilament correlates with outcome after aneurysmal subarachnoid Hemorrhage. Neurosci Lett.

[35] Lu AY, Damisah EC, Winkler EA, Grant RA, Eid T, Bulsara KR (2018). Cerebrospinal fluid untargeted metabolomic profiling of aneurysmal subarachnoid Hemorrhage: an exploratory study. Br J Neurosurg.

[36] Johansson C, Koskinen LD, Sjöberg RL, Lindvall P (2022). Serum levels of Myo-Inositol predicts clinical outcome 1 year after Aneurysmal Subarachnoid Hemorrhage. Neurosurgery.

[37] Bian L, Lin J, Liu Y, Lu J, Zhao X (2021). Copeptin and insulin-like growth factor-1 predict long-term outcomes after aneurysmal subarachnoid Hemorrhage: a large prospective cohort study. Clin Neurol Neurosurg.

[38] Zhang R, Liu Z, Zhang Y, Pei Y, He Y, Yu J, You C, Ma L, Fang F (2023). Improving the models for prognosis of aneurysmal subarachnoid Hemorrhage with the neutrophil-to-albumin ratio. Front Neurol.

[39] Provencio JJ, Swank V, Lu H, Brunet S, Baltan S, Khapre RV, Seerapu H, Kokiko-Cochran ON, Lamb BT, Ransohoff RM (2016). Neutrophil depletion after subarachnoid Hemorrhage improves memory via NMDA receptors. Brain Behav Immun.

[40] Suarez JI, Martin RH, Calvillo E, Dillon C, Bershad EM, Macdonald RL, Wong J, Harbaugh R (2012). The Albumin in Subarachnoid Hemorrhage (ALISAH) multicenter pilot clinical trial: safety and neurologic outcomes. Stroke.

[41] Zhang Y, Li L, Jia L, Li T, Di Y, Wang P, Deng H, Fan H, Li Y, Cheng X (2021). Neutrophil counts as promising marker for Predicting In-Hospital mortality in Aneurysmal Subarachnoid Hemorrhage. Stroke.

[42] Manda-Handzlik A, Demkow U. The brain entangled: the contribution of Neutrophil Extracellular traps to the Diseases of the Central Nervous System. Cells 2019, 8(12). 10.3390/cells8121477 PMC6953104 31766346

[43] Thanabalasuriar A, Scott BNV, Peiseler M, Willson ME, Zeng Z, Warrener P, Keller AE, Surewaard BGJ, Dozier EA, Korhonen JT (2019). Neutrophil extracellular traps confine Pseudomonas aeruginosa Ocular biofilms and restrict Brain Invasion. Cell Host Microbe.

[44] Vaibhav K, Braun M, Alverson K, Khodadadi H, Kutiyanawalla A, Ward A, Banerjee C, Sparks T, Malik A, Rashid MH (2020). Neutrophil extracellular traps exacerbate neurological deficits after traumatic brain injury. Sci Adv.

[45] Wang P, Zhang Y, Wang X, Peng L, Jia L, Li T, Chong W, Hai Y, You C, Fang F. Association between Serum Albumin and hospital-acquired Infections after Aneurysmal Subarachnoid Hemorrhage. Neurocrit Care 2021. 10.1007/s12028-021-01421-y 34970707

[46] Shang F, Zhao H, Cheng W, Qi M, Wang N, Qu X (2021). Predictive value of the serum albumin level on admission in patients with spontaneous subarachnoid Hemorrhage. Front Surg.

[47] Wang L, Li M, Xie Y, Xu L, Ye R, Liu X (2017). Preclinical efficacy of human albumin in subarachnoid Hemorrhage. Neuroscience.

[48] Xie Y, Guo H, Wang L, Xu L, Zhang X, Yu L, Liu Q, Li Y, Zhao N, Zhao N (2017). Human albumin attenuates excessive innate immunity via inhibition of microglial Mincle/Syk signaling in subarachnoid Hemorrhage. Brain Behav Immun.

[49] Xie Y, Liu W, Zhang X, Wang L, Xu L, Xiong Y, Yang L, Sang H, Ye R, Liu X (2015). Human albumin improves long-term behavioral sequelae after subarachnoid Hemorrhage through neurovascular remodeling. Crit Care Med.

[50] Ivens S, Kaufer D, Flores LP, Bechmann I, Zumsteg D, Tomkins O, Seiffert E, Heinemann U, Friedman A (2007). TGF-beta receptor-mediated albumin uptake into astrocytes is involved in neocortical epileptogenesis. Brain.

[51] Lodge KM, Vassallo A, Liu B, Long M, Tong Z, Newby PR, Agha-Jaffar D, Paschalaki K, Green CE, Belchamber KBR (2022). Hypoxia increases the potential for neutrophil-mediated endothelial damage in Chronic Obstructive Pulmonary Disease. Am J Respir Crit Care Med.

[52] Quint JK, Wedzicha JA (2007). The neutrophil in Chronic Obstructive Pulmonary Disease. J Allergy Clin Immunol.

[53] Zinellu E, Fois AG, Sotgiu E, Mellino S, Mangoni AA, Carru C, Zinellu A, Pirina P. Serum albumin concentrations in stable Chronic Obstructive Pulmonary Disease: a systematic review and Meta-analysis. J Clin Med 2021, 10(2). 10.3390/jcm10020269 PMC7828417 33450916

[54] Barkaway A, Rolas L, Joulia R, Bodkin J, Lenn T, Owen-Woods C, Reglero-Real N, Stein M, Vázquez-Martínez L, Girbl T (2021). Age-related changes in the local milieu of inflamed tissues cause aberrant neutrophil trafficking and subsequent remote organ damage. Immunity.

[55] Toyoshima K, Seino S, Tamura Y, Ishikawa J, Chiba Y, Ishizaki T, Fujiwara Y, Shinkai S, Kitamura A, Araki A (2022). Difference between physical fitness age based on physical function and chronological age is Associated with obesity, hyperglycemia, depressive symptoms, and low serum albumin. J Nutr Health Aging.

[56] Harrington D, D’Agostino RB, Sr., Gatsonis C, Hogan JW, Hunter DJ, Normand ST, Drazen JM, Hamel MB (2019). New guidelines for statistical reporting in the Journal. N Engl J Med.

