# Supplementary material for: Association between neutrophil-to-albumin ratio and long-term mortality of aneurysmal subarachnoid hemorrhage
Source: BMC Neurol. 2023 Oct 19;23:374. doi: 10.1186/s12883-023-03433-x (PMC10585913; doi:10.1186/s12883-023-03433-x)
Supplement: Supplementary file 1 — Supplementary Material 1 [file 12883_2023_3433_MOESM1_ESM.docx]

**Table S1 Univariate and multivariate cox regression analysis for long-term mortality of all patients**

| **Characteristics** | **Unadjusted** | | **Multivariable Regression Adjustment** | |
| --- | --- | --- | --- | --- |
|  | **HR (95% CI)** | ***P* value** | **HR (95% CI)** | ***P* value** |
| Demographics | | | | |
| Age | 1.03 (1.03-1.04) | <0.001 | 1.02 (1.01-1.03) | <0.001 |
| Male | 1.23 (1.08-1.38) | 0.007 | 1.16 (1.00-1.32) | 0.07 |
| Smoking |  |  |  |  |
| Current | Ref |  |  |  |
| Ever | 1.19 (0.79-1.60) | 0.39 |  |  |
| Never | 1.11 (0.73-1.49) | 0.58 |  |  |
| Alcohol abuse | 1.02 (0.83-1.20) | 0.86 |  |  |
| Systolic BP | 1.01 (1.01-1.01) | <0.001 | 1.00 (1.00-1.01) | 0.09 |
| Medical history | | | | |
| Hypertension | 1.24 (1.07-1.40) | 0.01 | 0.92 (0.75-1.10) | 0.37 |
| Diabetes | 1.43 (1.16-1.71) | 0.01 | 0.79 (0.48-1.10) | 0.14 |
| Coronary heart disease | 1.29 (0.87-1.72) | 0.24 |  |  |
| Chronic renal failure | 4.43 (3.85-5.00) | <0.001 | 2.53 (1.91-3.15) | 0.003 |
| COPD | 1.77 (1.53-2.00) | <0.001 | 1.08 (0.82-1.33) | 0.57 |
| Aneurysm characteristics | | | | |
| Posterior location | 1.23 (1.04-1.41) | 0.03 | 0.92 (0.72-1.12) | 0.42 |
| Size of aneurysm | 1.26 (1.17-1.34) | <0.001 | 1.17 (1.10-1.25) | <0.001 |
| Hemorrhagic characteristics | | | | |
| Fisher grade III-IV | 2.01 (1.80-2.23) | <0.001 | 1.35 (1.13-1.58) | 0.008 |
| Hunt & Hess grade IV-V | 5.56 (5.41-5.72) | <0.001 | 2.58 (2.39-2.77) | <0.001 |
| External ventricular drain | 3.95 (3.65-4.26) | <0.001 | 1.80 (1.48-2.13) | <0.001 |
| Treatment of aneurysms | | | | |
| No treatment | Ref |  |  |  |
| Clip | 0.28 (0.12-0.44) | <0.001 | 0.35 (0.18-0.52) | <0.001 |
| Coil | 0.28 (0.01-0.55) | <0.001 | 0.41 (0.13-0.69) | <0.001 |
| Biology | | | | |
| Glucose, mmol/L | 1.16 (1.14-1.18) | <0.001 | 1.07 (1.05-1.10) | <0.001 |
| NAR (<0.15) | Ref |  |  |  |
| NAR (0.15-0.20) | 1.55 (1.19-2.02) | 0.001 | 1.30 (0.97-1.73) | 0.08 |
| NAR (0.20-0.28) | 1.92 (1.49-2.48) | <0.001 | 1.37 (1.03-1.82) | 0.03 |
| NAR (>0.28) | 4.06 (3.22-5.13) | <0.001 | 1.74 (1.30-2.32) | <0.001 |

NAR, neutrophil-to-albumin ratio; SBP, systolic blood pressure; CHD, coronary heart disease; COPD, chronic obstructive pulmonary disease; CRF, chronic renal failure; HR, hazard rate; CI, confidence interval.

**Table S2 Univariate and multivariate cox regression analysis for long-term mortality of survivors at discharge**

| **Characteristics** | **Unadjusted** | | **Multivariable Regression Adjustment** | |
| --- | --- | --- | --- | --- |
|  | **HR (95% CI)** | ***P* value** | **HR (95% CI)** | ***P* value** |
| Demographics | | | | |
| Age | 1.04 (1.03-1.05) | <0.001 | 1.03 (1.02-1.04) | <0.001 |
| Male | 1.22 (1.05-1.40) | 0.02 | 1.21 (1.02-1.39) | 0.05 |
| Smoking |  |  |  |  |
| Current | Ref |  |  |  |
| Ever | 1.27 (0.79-1.74) | 0.33 |  |  |
| Never | 1.17 (0.72-1.62) | 0.50 |  |  |
| Alcohol abuse | 1.03 (0.82-1.25) | 0.76 |  |  |
| Systolic BP | 1.01 (1.01-1.02) | <0.001 | 1.00 (1.00-1.01) | 0.008 |
| Medical history | | | | |
| Hypertension | 1.24 (1.04-1.43) | 0.03 | 0.92 (0.71-1.12) | 0.39 |
| Diabetes | 1.43 (1.11-1.76) | 0.03 | 0.83 (0.47-1.19) | 0.31 |
| Coronary heart disease | 1.40 (0.93-1.87) | 0.16 |  |  |
| Chronic renal failure | 5.30 (4.64-5.96) | <0.001 | 2.93 (2.22-3.65) | 0.003 |
| COPD | 1.91 (1.64-2.18) | <0.001 | 1.06 (0.77-1.35) | 0.70 |
| Aneurysm characteristics | | | | |
| Posterior location | 1.24 (1.02-1.45) | 0.06 | 0.95 (0.72-1.18) | 0.66 |
| Size of aneurysm | 1.26 (1.16-1.35) | <0.001 | 1.19 (1.10-1.28) | <0.001 |
| Hemorrhagic characteristics | | | | |
| Fisher grade III-IV | 1.65 (1.42-1.89) | <0.001 | 1.19 (0.95-1.43) | 0.15 |
| Hunt & Hess grade IV-V | 4.55 (4.36-4.74) | <0.001 | 2.30 (2.07-2.53) | <0.001 |
| External ventricular drain | 4.20 (3.83-4.57) | <0.001 | 2.17 (1.78-2.57) | <0.001 |
| Treatment of aneurysms | | | | |
| No treatment | Ref |  |  |  |
| Clip | 0.32 (0.14-0.51) | <0.001 | 0.40 (0.20-0.60) | <0.001 |
| Coil | 0.30 (0.02-0.61) | <0.001 | 0.42 (0.09-0.74) | <0.001 |
| Biology | | | | |
| Glucose, mmol/L | 1.14 (1.12-1.17) | <0.001 | 1.06 (1.03-1.10) | <0.001 |
| NAR (<0.14) | Ref |  |  |  |
| NAR (0.14-0.20) | 1.63 (1.21-2.19) | 0.001 | 1.34 (0.97-1.85) | 0.08 |
| NAR (0.20-0.27) | 1.96 (1.47-2.62) | <0.001 | 1.51 (1.09-2.09) | 0.01 |
| NAR (>0.27) | 3.28 (2.50-4.28) | <0.001 | 1.55 (1.11-2.15) | 0.009 |

NAR, neutrophil-to-albumin ratio; SBP, systolic blood pressure; CHD, coronary heart disease; COPD, chronic obstructive pulmonary disease; CRF, chronic renal failure; HR, hazard rate; CI, confidence interval.

**Table S3 Associations between NAR levels after logarithm transformation and mortality in all patients and discharged survivors**

| **Outcomes** | **Unadjusted** | | **Multivariable Regression Adjustment** | |
| --- | --- | --- | --- | --- |
|  | **Unadjusted HR** | ***P* value** | **Adjusted HR** | ***P* value** |
| Mortality before discharge | 7.75 (5.42-11.08) | <0.001 | 2.82 (1.87-4.26) | <0.001 |
| 1-year mortality | 4.45 (3.66-5.42) | <0.001 | 2.07 (1.67-2.57) | <0.001 |
| Long-term mortality | 3.23 (2.75-3.79) | <0.001 | 1.78 (1.49-2.12) | <0.001 |
| 1-year mortality of discharged survivors | 3.48 (2.72-4.45) | <0.001 | 1.84 (1.40-2.43) | <0.001 |
| Long-term mortality of discharged survivors | 2.52 (2.10-3.03) | <0.001 | 1.55 (1.26-1.90) | <0.001 |

NAR, neutrophil-to-albumin ratio; HR, hazard rate; CI, confidence interval.

**Table S4 Categorical net reclassification improvement of NAR vs other inflammatory biomarkers at different time points**

| **Biomarkers** | **cNRI^1^ (95% CI)** | ***P* value** | **cNRI^2^ (95% CI)** | ***P* value** | **cNRI^3^ (95% CI)** | ***P* value** |
| --- | --- | --- | --- | --- | --- | --- |
| NAR vs PAR | 0.13 (0.09-0.17) | <0.001 | 0.18 (0.14-0.22) | <0.001 | 0.08 (0.05-0.11) | <0.001 |
| NAR vs NLR | 0.15 (0.11-0.20) | <0.001 | 0.18 (0.14-0.22) | <0.001 | 0.08 (0.06-0.11) | <0.001 |
| NAR vs PLR | 0.25 (0.20-0.29) | <0.001 | 0.18 (0.14-0.22) | <0.001 | 0.08 (0.06-0.11) | <0.001 |
| NAR vs MLR | 0.25 (0.20-0.29) | <0.001 | 0.18 (0.15-0.22) | <0.001 | 0.08 (0.06-0.11) | <0.001 |
| NAR vs SII | 0.25 (0.20-0.29) | <0.001 | 0.18 (0.15-0.22) | <0.001 | 0.08 (0.06-0.11) | <0.001 |

NAR, neutrophil-to-albumin ratio; PAR, platelet-to-albumin ratio; NLR, neutrophil-to-lymphocyte ratio; PLR, platelet-to-lymphocyte ratio; MLR, monocyte-to-lymphocyte ratio; SII, systemic immune inflammation index; cNRI, categorical net reclassification improvement

^1^ cNRI at 6 months

^2^ cNRI at 1 year

^3^ cNRI at 3 years

**Figure S1 Calculation methods for the different combinations of inflammatory factors.**


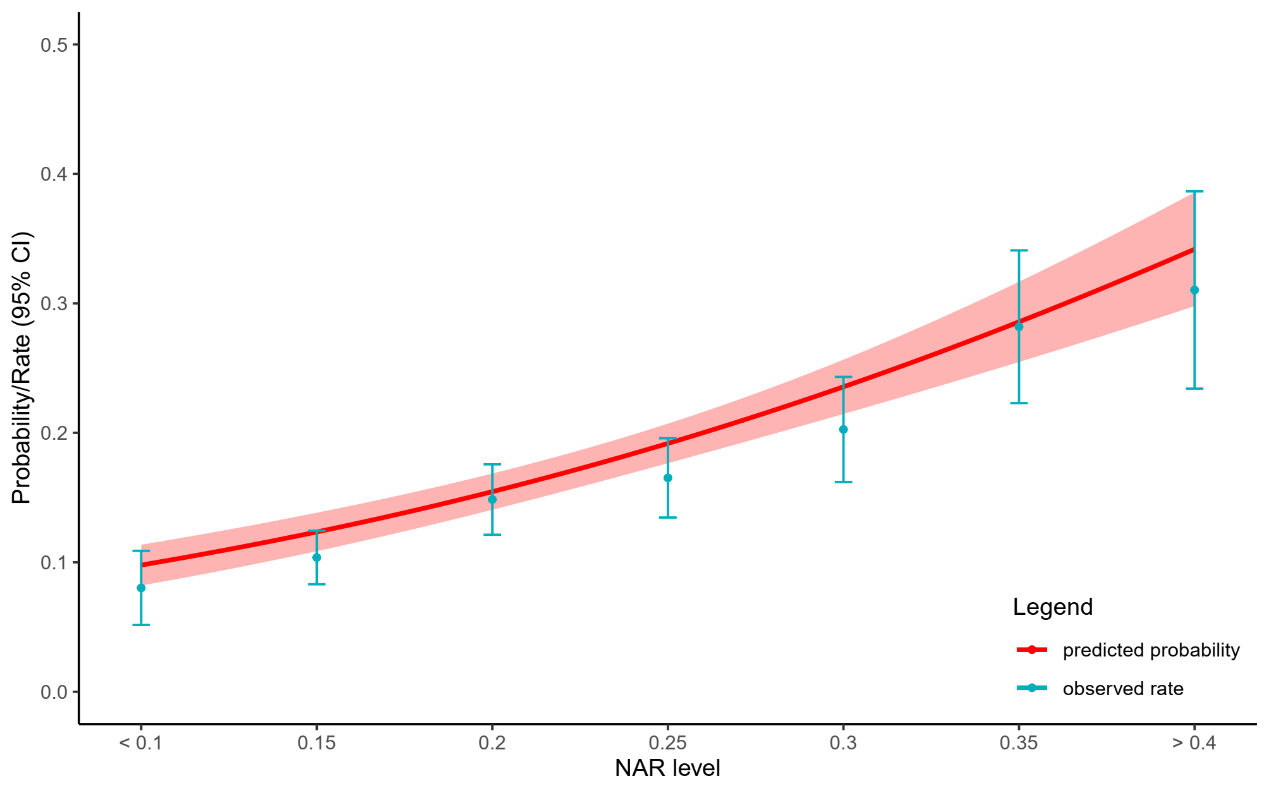


1.
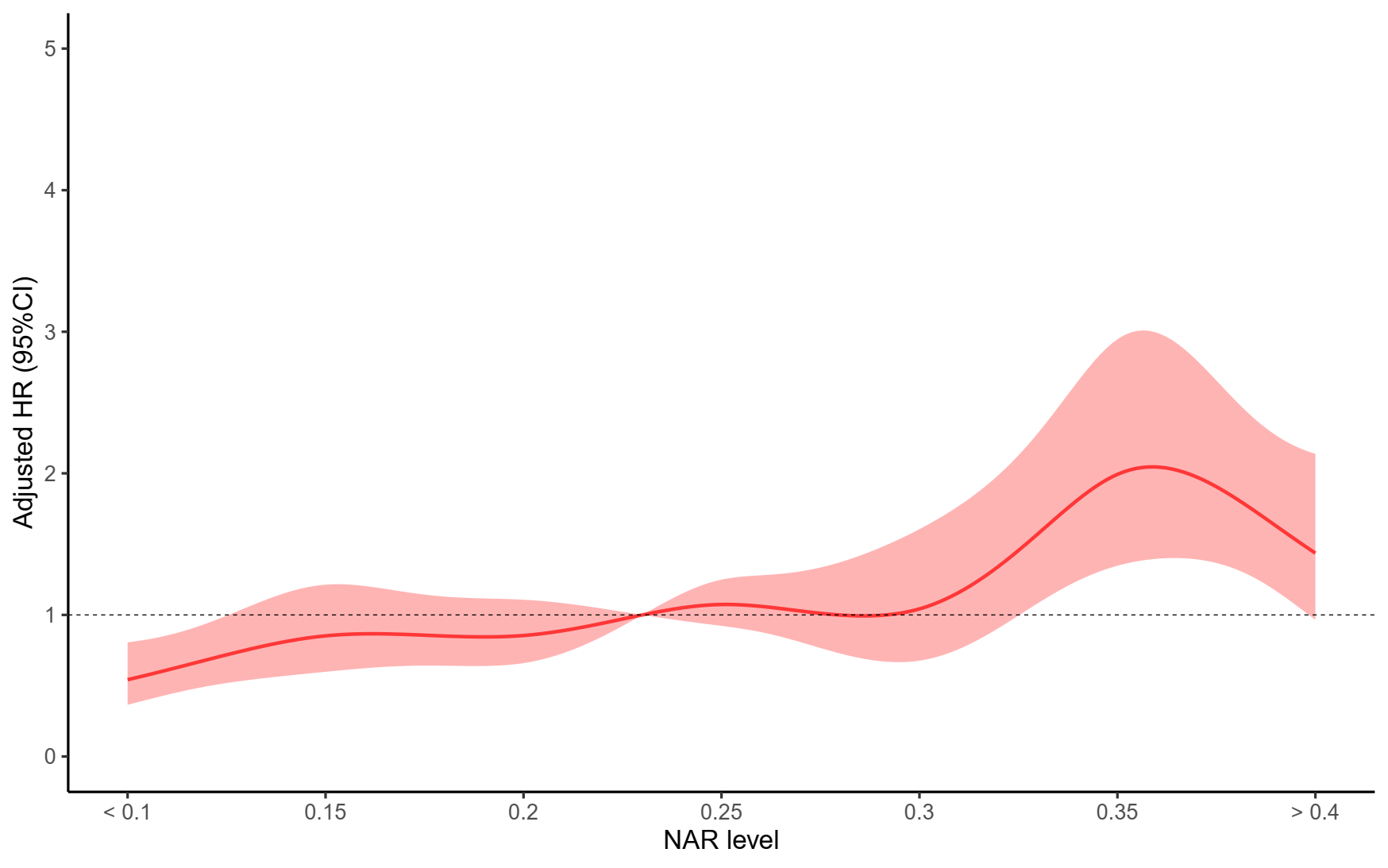


**Figure S2 Relationship between NAR and long-term mortality in survivors at discharge. Predicted probabilities and observed rate of long-term mortality (a). Adjusted HR and 95% CI are shown for each 0.05 change (b). NAR, neutrophil-to-albumin ratio; HR, hazard ratio; CI, confidence interval.**
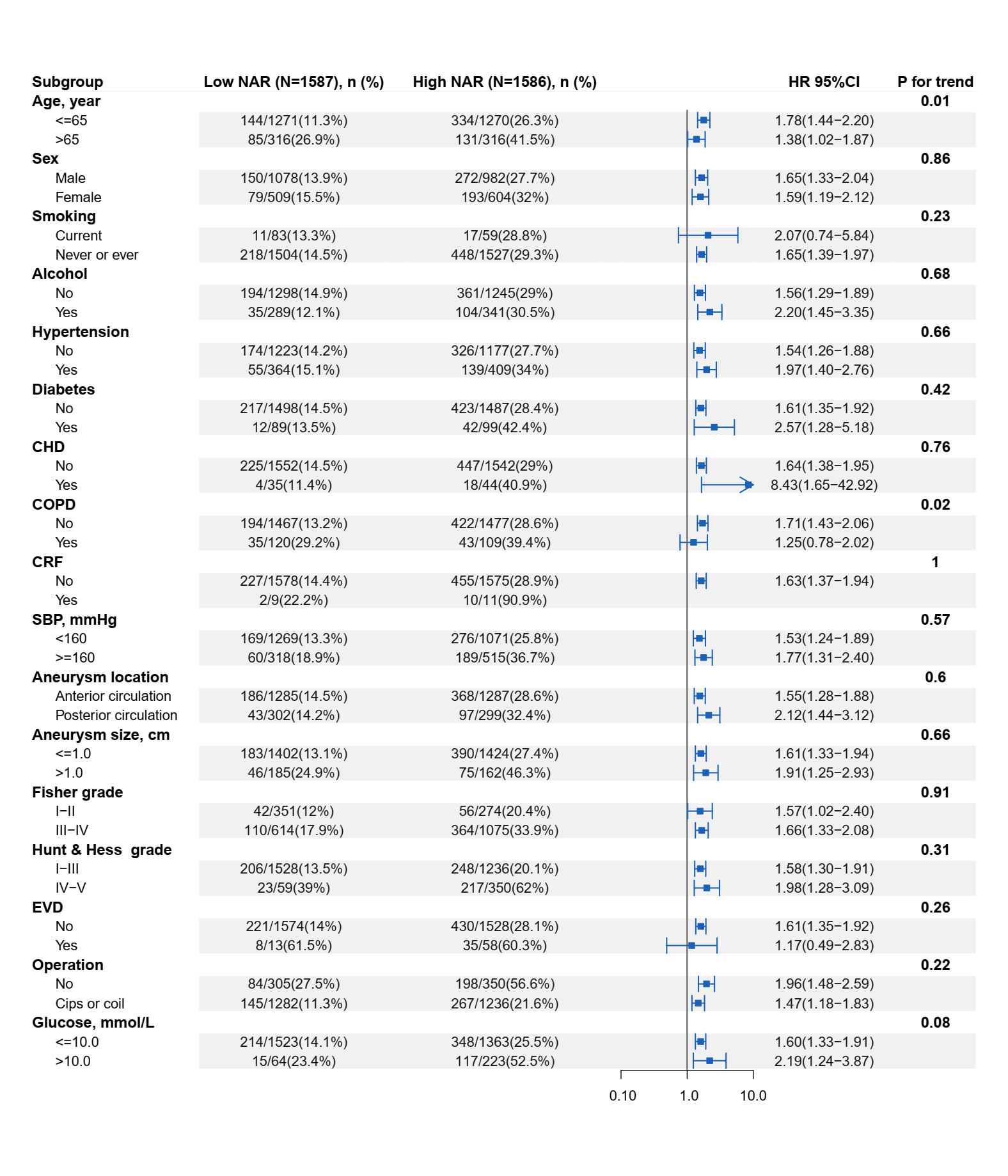
 **Figure S3 Subgroup analysis of the association between NAR levels and long-term mortality with a multivariate Cox regression model.Low NAR: ≤ 0.27; high NAR: > 0.27.**
